# Preoperative knee joint hypothermia reduces inflammation and recovery time and increases range of motion after total knee arthroplasty: A randomized controlled trial

**DOI:** 10.1002/ksa.12756

**Published:** 2025-07-02

**Authors:** Leonardo Pieri, Filippo Leggieri, Dimitri Bartoli, Marco Ponti, Chiara Caparrini, Andrea Baldini

**Affiliations:** ^1^ Istituto Fiorentino Di Cura E Assistenza (IFCA) Florence Italy; ^2^ Department of Clinical Orthopedics University of Florence Florence Italy

**Keywords:** cryocompression therapy, hypothermia, inflammation markers, preoperative cryotherapy, rehabilitation, total knee arthroplasty

## Abstract

**Purpose:**

Cryotherapy modulates synovial fluid composition by reducing inflammatory mediators and altering metabolite concentrations, resulting in both anti‐inflammatory and anti‐oxidative effects. The aim was to investigate whether preoperative cryocompression would (1) maintain deep tissue hypothermia during the surgical procedure, (2) attenuate inflammatory response and tissue damage and (3) improve post‐operative outcomes.

**Methods:**

The study enroled patients undergoing total knee arthroplasty (TKA) surgery who were randomly assigned to either the control group (*n* = 50) receiving post‐operative cryocompression therapy, or the intervention group (*n* = 50) receiving both preoperative and post‐operative cryocompression. Exclusion criteria: coagulation/psychiatric disorders. Inclusion criteria: monolateral primary Kellgren–Lawrence Grade IV knee osteoarthritis. Multiple outcomes, including patient‐reported outcomes (PROs), discharge milestones, cutaneous/osseous temperature and inflammatory biomarkers, were assessed. The primary end point was the difference in inflammatory markers across cohorts, while secondary end points included differences in pain, patient‐reported outcome measures (PROMs) and range of motion (ROM). Chi‐square test was used for categorical variables and the Mann–Whitney *U*‐test for continuous variables. The minimally clinically important difference was calculated using the 0.5 SD approach. Linear mixed‐effects models analyzed the differences in inflammatory markers between the cohorts over time. The alpha value was set at 0.05.

**Results:**

Differences between the contralateral and the operated knee skin temperatures (*Z* = −4.5, *p* < 0.001), as well as between the contralateral knee skin temperature and the operated knee bone temperature (*Z* = −4.9, *p* < 0.001) were found. (2) Erythrocyte sedimentation rate was lower in the intervention group on post‐operative Days 1 and 2 compared to the control group. Fibrinogen had a greater increase from preoperative to post‐operative in the control group (*U* = 746.5, *p* = 0.018). (3) Higher ROM (*p* = 0.001) and shorter time to reach the rehabilitation milestones to discharge (*p* = 0.007) were found in the intervention group.

**Conclusions:**

Preoperative cryocompression therapy before TKA might reduce early post‐operative inflammation, accelerate rehabilitation milestones, and improve early ROM.

**Levels of Evidence:**

Level IV.

**Abbreviations**
BMI: body mass index
CI: confidence interval
CRP: C‐reactive protein
ESR: erythrocyte sedimentation rate
IL‐1β: interleukin‐1 beta
IL‐6: Interleukin‐6
IQR: interquartile range
KSS: Knee Society Score
MC: medially congruent
MCID: minimally clinically important difference
OA: osteoarthritis
OKS: Oxford Knee Score
ROM: range of motion
TKA: total knee arthroplasty
VAS: visual analogue scale

## INTRODUCTION

Cryotherapy in the form of ice and compression wraps has been recognized as an effective treatment following total knee arthroplasty (TKA) for decades [5, 17, 19], and it works through multiple physiological mechanisms. Cold exposure triggers vasoconstriction [35], which reduces local metabolism and inflammation [18], decreasing capillary permeability and oedema [35], while simultaneously activating pain control pathways [8] and creating an environment that inhibits bacterial growth [2]. When combined with compression, the cold penetrates more effectively into deeper tissues [1, 16, 36] and helps prevent fluid accumulation by reducing vascular‐tissue pressure gradients [12, 28].

The cooling and compression affect the joint temperatures, correlating with lower prostaglandin E2, thus decreasing the inflammation of the synovia [30]. Cryotherapy also reduces synovial inflammatory mediators in arthritic knees, specifically interleukin (IL)‐6, IL‐1 beta and vascular endothelial growth factor [9].

While cryotherapy has been shown to reduce narcotic requirements, improve the 6‐min walk test, and enhance patient satisfaction after TKA [32, 33, 34], its preoperative application in TKA surgery remains unexplored.

Given that TKA surgery triggers a massive inflammatory response, this study hypothesized that performing TKAs under profound joint hypothermia would attenuate post‐operative inflammation, thereby facilitating enhanced recovery.

Therefore, the aim of this study was to investigate whether preoperative cryocompression, administered for 2 h prior to surgery using the Zamar device until surgical field preparation, would (1) maintain deep tissue hypothermia during the surgical procedure, (2) attenuate inflammatory response and tissue damage and (3) improve post‐operative outcomes, specifically pain, swelling and functional recovery parameters compared to post‐operative cryocompression alone.

## MATERIALS AND METHODS

A single‐centre, parallel‐group, randomized controlled trial was conducted between June 2021 and May 2022. The study enroled 100 patients undergoing TKA surgery who were randomly assigned to either the control group (*n* = 50) receiving post‐operative cryocompression therapy or the intervention group (*n* = 50) receiving both preoperative and post‐operative cryocompression therapy. The study followed CONSORT guidelines [26].

Patients with coagulation disorders, psychiatric conditions, known allergies to medications used in either protocol, or intolerance to cryocompression therapy, and those without written informed consent were excluded. The inclusion criterion was unilateral primary bone‐on‐bone Kellgren–Lawrence Grade IV knee osteoarthritis (OA) [15]. The study cohort (*N* = 100) included predominantly female participants (*n* = 73, 73%), with a median age of 74 years (interquartile range [IQR] = 68–77) and a median body mass index (BMI) of 28.4 (IQR = 24.8–31.6).

### Sample size

The sample size of 100 participants (50 per group) was calculated using G*Power software. Given the novel nature of preoperative cryotherapy in TKA, with no prior studies available for reference, a medium effect size (0.5) was assumed for detecting differences in inflammatory markers between groups. With a significance level of 5% and power of 80%, this calculation supported the final sample size of 50 patients per group.

### Randomization and blinding

An independent statistician generated the random allocation sequence using computer‐generated random numbers in four blocks to ensure balanced group sizes. A research fellow not involved in the assessment enroled participants and assigned them to interventions. Due to the nature of the intervention, complete blinding was not feasible; however, outcome assessors and the statistician were blinded to group allocation. All the physiotherapy and internal medicine personnel were blinded.

### Interventions

**Figure 1:**
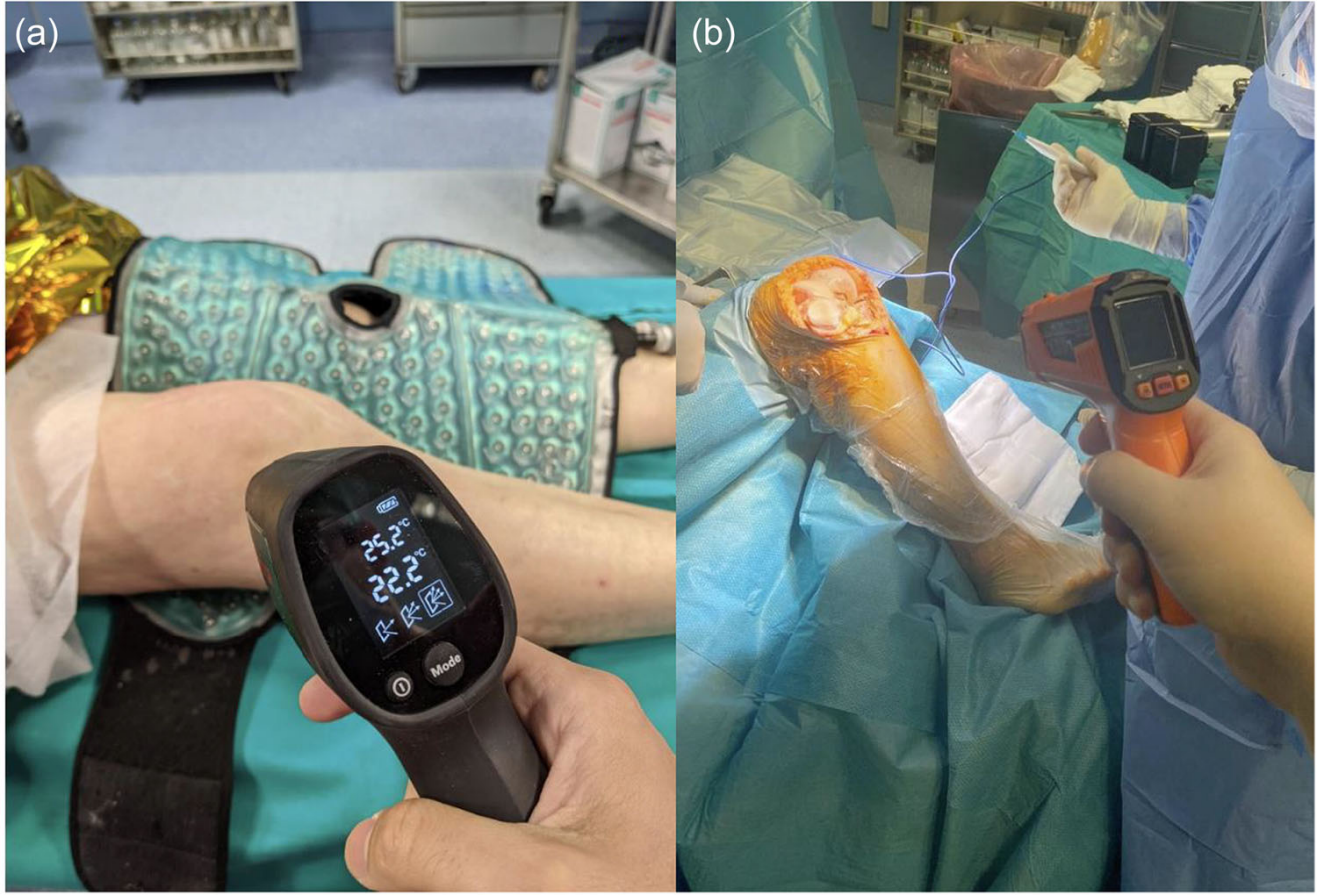
The image shows the skin and bone temperature assessment using the infrared thermometer. (a) Superficial skin temperature was measured before incision. (b) Following arthrotomy, bone temperature was assessed to confirm the effect of preoperative cryotherapy on deep tissues.

All surgical procedures were performed using a standardized technique by the same surgical team. In the intervention group, patients received preoperative cryocompression therapy at 5°C for 1.5 h prior to surgery using the brace from the device Zamar ZT Clinic PRO (Zamar Medical S.r.l.) (Figure 1) [11], which remained in place until surgical field preparation. Post‐operatively, patients began full weight‐bearing mobilization once the selective spinal anaesthesia in the operated leg wore off. Each patient received daily cryotherapy sessions lasting 4–5 h, continuing this protocol for 2 weeks. The control group (*n* = 50) received only post‐operative cryotherapy with the Zamar ZT Clinic PRO device (Zamar Medical S.r.l.) following the same protocol of 4–5 h daily for 2 weeks. All post‐operative cryotherapy sessions in both groups were administered using the same equipment and settings.

All the surgical procedures were performed through a midline skin incision with a medial parapatellar arthrotomy. A pneumatic tourniquet was inflated during the bone preparation and the cement fixation phases [24]. All patients received the same perioperative protocol of tranexamic acid and corticosteroids [3]. The Persona® Medially Congruent (MC) Total Knee System (Zimmer Biomet) was implanted in all cases following a tibia‐first personalized alignment approach. Both groups followed standard post‐operative care protocols, including pain management and rehabilitation programmes. All patients followed the same deep vein thrombosis prophylaxis protocol.

### Outcome measurement

Multiple outcome measures were assessed at predefined time points (Table 1). Clinical outcomes included the Knee Society Score (KSS) [13], the Oxford Knee Score [21] and range of motion (ROM). Physical measurements included knee circumference assessment (cm), comparing the circumference at specific post‐operative days and basal (∆ knee circumference), skin and bone temperature (T°) (Figure 1), visual analogue scale (VAS) scores both at rest and after physiotherapy. Laboratory parameters, including erythrocyte sedimentation rate (ESR), C‐reactive protein (CRP) and Fibrinogen, were monitored to assess inflammatory and coagulation status. All complications were recorded. Rehabilitation milestones for hospital discharge criteria included the ability to independently transfer in and out of the bed, the chair, and the toilet seat; independently ambulate approximately 150 m; independently negotiate stairs; and be independent with a home exercise programme and activities of daily living. The primary end point was the difference in inflammatory marker results during the post‐operative assessment across the cohorts. Secondary end points were the differences in pain scores (VAS), patient‐reported outcome measures (PROMs) and ROM across the cohorts at the final follow‐up. Knee circumference assessment was assessed with the same ordinary tape measure and recorded by the same clinician for all the measurements [14]. Skin and bone temperature were assessed with the same infrared thermometer by the same clinician for all the measurements [27]. Blood samples collected for laboratory parameter analysis were processed by the same institutional laboratory.

**Table 1:** Timeline of outcome measures assessments.

	Before TKA	Intraoperatively	After 4 h from TKA	After 1 day from TKA	After 2 days from TKA	After 20 days from TKA
Knee Society Score	✓	x	x	x	✓	✓
Oxford Knee Score	✓	x	x	x	x	x
Proximal leg circumference at the top of the patella (in extension)	✓	x	✓	✓	✓	✓
Skin knee temperature (affected and contralateral)	✓	x	x	x	x	x
Bone knee temperature (affected and contralateral)	x	✓	x	x	x	x
VAS at rest	x	x	✓	✓	✓	x
VAS following the physiotherapy	x	x	✓	✓	✓	x
Complications	x	x	✓	✓	✓	✓
Time to rehab milestones (every 0.5 days)	x	x	x	x	✓	x
ESR, CRP and fibrinogen	x	x	x	✓	✓	x

Abbreviations: CRP, C‐reactive protein; ESR, erythrocyte sedimentation rate; TKA, total knee arthroplasty; VAS, visual analogue scale.

### Ethics

The trial protocol was approved by the Institutional Ethics Committee of Giomi Innovation and Research (Protocol No. 5/21/CTS).

### Data analyses

Descriptive statistics were calculated for all variables. Following Shapiro–Wilk results (*p* < 0.05), associations between categorical variables were assessed using the chi‐square test, while differences between continuous variables were analyzed using the Mann–Whitney *U*‐test for independent variables and the Wilcoxon test for dependent variables; Cliff's delta was measured for effect size calculation. Pearson's correlation coefficient was used to assess associations between continuous variables. The minimally clinically important difference (MCID) was calculated using the 0.5 standard deviation approach, specifically by multiplying the standard deviation of the baseline measurements for the outcome measure by 0.5. Linear mixed‐effects models were employed to analyse differences in inflammatory markers between the cohorts over time. All analyses were performed using R Software (R version 4.1.0). Statistical significance was set at *p* < 0.05.

## RESULTS

**Figure 2:**
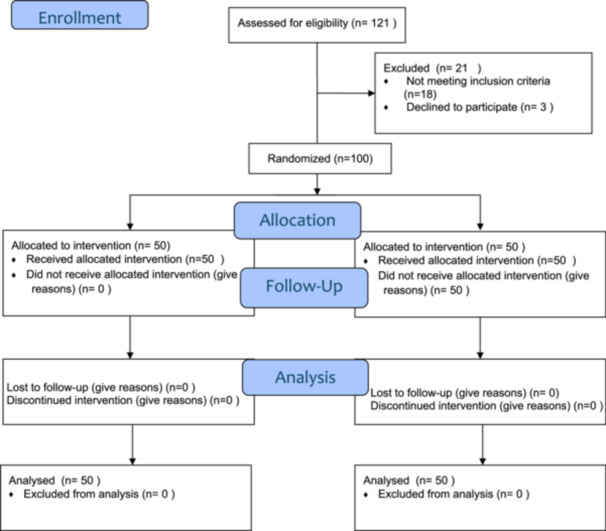
Flow diagram according to CONSORT 2010 guidelines.

Between June 2021 and May 2022, 121 patients were assessed for eligibility. Figure 2 shows the patient selection flowchart. All participants received the allocated intervention and completed the 20‐day follow‐up, with no losses to follow‐up or protocol deviations. Demographics and baseline characteristics are presented in Table 2. Comprehensive post‐operative outcomes and between‐group comparisons are presented in Table 3.

**Table 2:** Baselines of the included patients across the cohorts.

	Intervention group		Control group		Mann–Whitney *U*‐test	Effect size	
	Median	IQR	Median	IQR	*p*	Cliff's delta	95% CI
Age	73	66–77	75.5	69.5–79.2	0.13	−0.17	[−0.38 to 0.054]
Women sex (*n* [%])	30 [60]		43 [86]		0.003		
Right side (*n* [%])	30 [60]		27 [54]		0.54		
BMI	28.5	25–31.2	27.3	24.1–33.4	0.98	0.002	[−0.22 to 0.22]
Maximum flexion (°)	110	103.7–120	110	100–120	0.28	0.12	[−0.10 to 0.34]
OKS	28	24–31	27	24–30	0.63	0.057	[−0.17 to 0.28]
KSS	57	50–65	55	48–60	0.29	0.13	[−0.11 to 0.36]
CRP	2.1	1.07–3.6	2.5	1.3–4.4	0.31	−0.11	[−0.32 to 0.10]
Fibrinogen	457	410–528	521	470–559	<0.001	−0.39	[−0.576 to −0.166]

Abbreviations: 95% CI, 95% confidence interval; BMI, body mass index; CRP, C‐reactive protein; IQR, interquartile range; KSS, Knee Society Score; OKS, Oxford Knee Score.

**Table 3:** Demographic and outcome data across the cohorts with Hodges–Lehmann estimates, effect sizes and 95% confidence intervals (CIs).

	Intervention group		Control group		Mann–Whitney *U*‐test	Effect size	
	Median	IQR	Median	IQR	*p*	Cliff's delta	95% CI
Post‐operative Day 0							
VAS before cryotherapy	0	0–0	0	0–0	0.9	0.008	[−0.12 to 0.14]
VAS after cryotherapy	0	0–0	0	0–0	0.4	−0.046	[−0.11 to 0.02]
Skin T° before cryotherapy (affected)	35.9	35.57–36.3	36.1	35.67–36.4	0.2	−0.132	[−0.34 to 0.092]
Skin T° before cryotherapy (contralateral)	36	35.8–36.3	36.1	35.67–36.3	0.9	0.014	[−0.21 to 0.23]
Knee circumference (affected)	44	41.7–47	44	42.87–47	0.4	−0.083	[−0.3 to 0.14]
Knee circumference (contralateral)	42	39.7–44.25	42	39.37–44.62	0.8	−0.03	[−0.26 to 0.20]
Post‐operative Day 1							
VAS before cryotherapy	0	0–0	0	0–0	0.6	0.039	[−0.11 to 0.19]
VAS after cryotherapy	0	0–0	0	0–0	0.6	−0.027	[−0.11 to 0.065]
Skin T° before cryotherapy (affected)	36.2	35.9–36.6	36.5	36.1–36.8	0.06	−0.21	[−0.41 to 0.002]
Skin T° before cryotherapy (contralateral)	36.2	36–36.5	36.3	36.2–36.6	0.1	−0.18	[−0.41 to 0.061]
Knee circumference (affected)	45	42–48	45	43–48.25	0.7	−0.043	[−0.26 to 0.18]
	1	0–2	0.75	0–1	0.5	0.066	[−0.15 to 0.28]
Knee circumference (contralateral)	42	40.37–45	42	39.87–45	0.8	0.028	[−0.20 to 0.26]
∆ Circumference contralateral	0	0–1	0	0–0	0.04*	0.20	[0–0.39]
ESR	6	2–12.25	14	6–22.5	0.001*	−0.37	[−0.56 to −0.13]
CRP	16.4	9.02–39.55	16.5	11.5–26.55	0.99	0.002	[−0.23 to 0.23]
Fibrinogen	454	421–516.5	497	433–559	0.023	−0.26	[−0.45 to −0.045]
D‐dimer	1087	644–1751	943	566–1405	0.33	0.11	[−0.12 to 0.34]
Post‐operative Day 2							
VAS before cryotherapy	0	0–0.2	0	0–0	0.23	0.094	[−0.064 to 0.24]
VAS after cryotherapy	0	0–0	0	0–0	0.48	−0.038	[−0.14 to 0.07]
Skin T° before cryotherapy (affected)	36.5	36.2–36.8	36.5	36.2–36.8	0.62	−0.058	[−0.28 to 0.17]
Skin T° before cryotherapy (contralateral)	36.3	36.1–36.5	36.4	36.2–36.5	0.25	−0.13	[−0.36 to 0.096]
Knee circumference (affected)	45.5	42.5–48.2	45.5	43.2–49	0.53	−0.073	[−0.28 to 0.15]
∆ Circumference affected	1	0–2	1	0.5–2	0.85	0.022	[−0.19 to 0.24]
Knee circumference (contralateral)	42	40.7–45	42	39.8–45	0.83	0.026	[−0.21 to 0.25]
∆ Circumference contralateral	0	0–1	0	0–0.5	0.10	0.18	[−0.036 to 0.38]
ESR	16	7–28	25	15–38.2	0.032	−0.26	[−0.48 to −0.019]
CRP	58.3	29.7–88.9	52.1	29.1–77.7	0.57	0.069	[−0.17 to 0.30]
Fibrinogen	596	545–638	598.5	544.2–644.5	0.99	−0.002	[−0.23 to 0.23]
D‐dimer	1072	779–1454	924	668–1302.5	0.17	0.16	[−0.07 to 0.39]
Post‐operative Day 3							
VAS before cryotherapy	0	0–0	0	0–0	0.22	0.076	[−0.02 to 0.17]
VAS after cryotherapy	0	0–0	0	0–0	0.31	−0.029	[−0.032 to −0.027]
Skin T° before cryotherapy (affected)	36.5	36.1–36.7	36.4	36.2–36.6	0.94	0.011	[−0.25 to 0.27]
Skin T° before cryotherapy (contralateral)	36.3	36.1–36.5	36.4	36.1–36.5	0.28	−0.15	[−0.42 to 0.13]
Knee circumference (affected)	45	4247.2	47	44–49	0.073	−0.24	[−0.47 to 0.012]
∆ Circumference affected	1	0–2	1.7	1–2.5	0.15	−0.19	[−0.43 to 0.069]
Knee circumference (contralateral)	42	41–44	43	40–45	0.65	−0.06	[−0.33 to 0.21]
∆ Circumference contralateral	0	0–0.75	0	0–0.5	0.72	0.047	[−0.20 to 0.29]
KSS	69	60–74	69	63–73	0.45	0.088	[−0.15 to 0.31]
Discharge day	1	1–1.5	1.2	1–1.5	0.082	−0.18	[−0.37 to 0.022]
Discharge and post‐operative Day 20							
Knee circumference (affected)	45	43–47.2	45	43–47.2	0.97	−0.004	[−0.23 to 0.22]
∆ Circumference affected	1	−0.6 to 2.1	1	−1 to 2	0.67	0.049	[−0.18 to 0.27]
Knee circumference (contralateral)	43	41–45	43	40–45	0.30	0.12	[−0.11 to 0.35]
∆ Circumference contralateral	1	0–2	0	0‐1	0.13	0.18	[−0.057 to 0.40]
OKS	27	23–28	26	22–28	0.30	0.12	[−0.097 to 0.33]
KSS	77	70–87	74.5	71–78	0.085	0.20	[−0.029 to 0.41]
Maximum flexion (°)	112.7 ± 8.9^a^	110–120	105.9 ± 10.1^a^	100–110	0.001*	0.37	[0.15–0.55]

Abbreviations: 95% CI, 95% confidence interval; ∆ circumference, comparative between circumference at specific post‐operative days and basal; CRP, C‐reactive protein; ESR, erythrocyte sedimentation rate; KSS, Knee Society Score; OKS, Oxford Knee Score; ROM, range of motion; VAS, visual analogue scale.

a Chi‐squared.

* *p* < 0.05.

### Effectiveness of cryotherapy on deep tissue hypothermia

In the intervention group, preoperative cryocompression therapy resulted in both lower skin and bone temperatures compared to the contralateral knee. Specifically, the median skin temperature of the operated knee just prior to skin incision reached 15.7°C (IQR = 13.0–18.6°C), while the bone temperature after arthrotomy was 21.4°C (IQR = 19–22.0°C). These values were significantly different from the contralateral knee (*Z* = −4.5, *p* < 0.001 for skin temperature; *Z* = −4.9, *p* < 0.001 for bone temperature).

### Attenuation of inflammatory response and tissue damage

**Figure 3:**
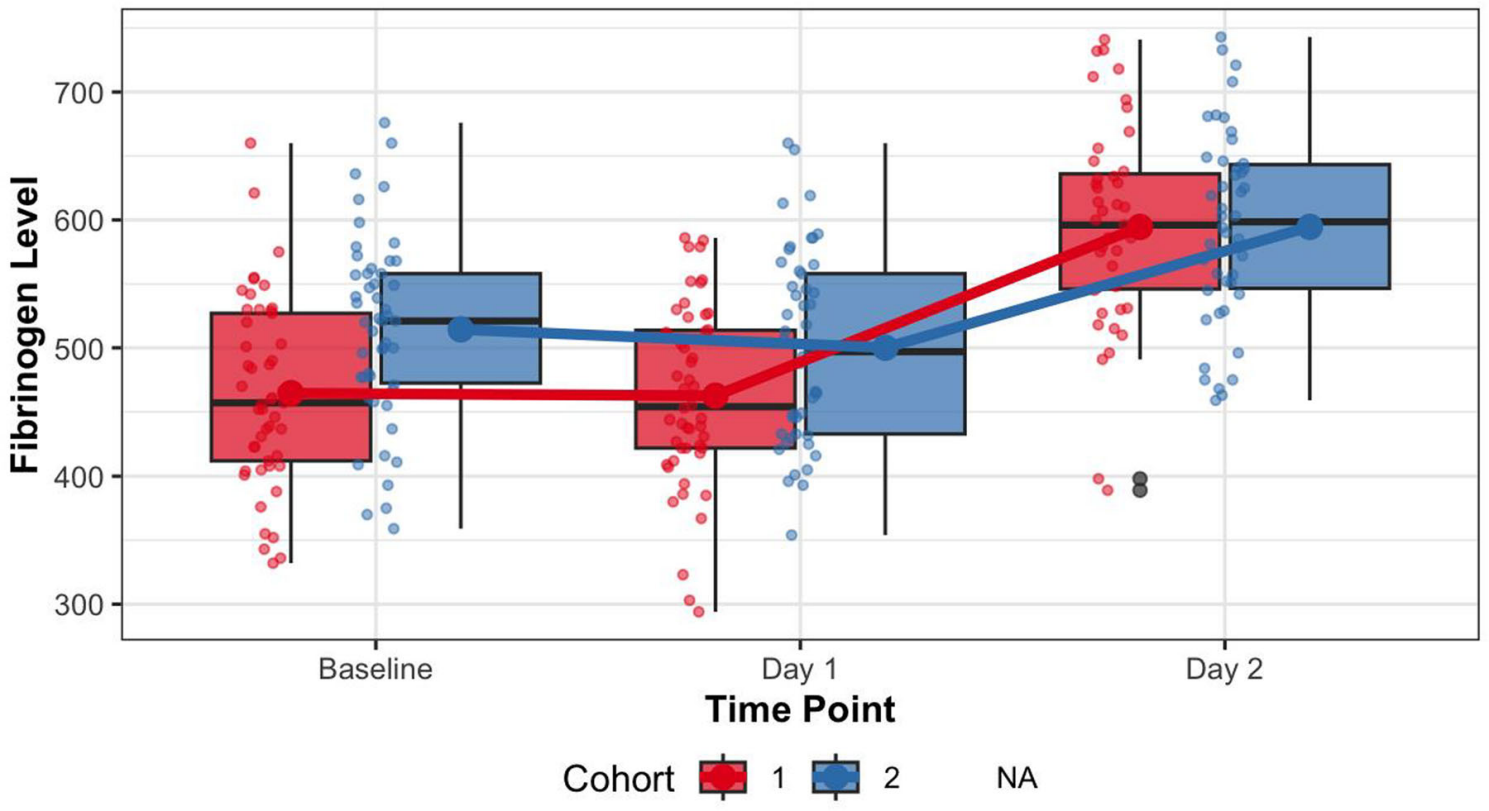
Temporal changes in fibrinogen levels stratified by cohort.

ESR was lower in the intervention group both on post‐operative Days 1 and 2 compared to the control group (Table 3), showing similar increases from preoperative to post‐operative Day 2 (*U* = 925.5, 0.88, Hodges–Lehmann = 0, 95% confidence interval [CI] = −5 to 4, effect size = 0.016). Preoperative fibrinogen levels showed differences between groups (Table 3). To account for these baseline differences, the variation of the fibrinogen was analyzed, revealing a greater increase from preoperative to post‐operative Day 2 in the intervention group (*U* = 746.5, *p* = 0.018, Hodges–Lehmann = 4, 95% CI = 1.5–6.8, effect size = 0.491) (Figure 3). Mixed‐effects model analysis on inflammatory markers showed different findings. CRP levels demonstrated a significant increase over time (*β* = 37.3, *p* < 0.001) regardless of group. The mixed‐effects model revealed that, although the intervention group showed a trend towards less CRP increase compared to the control group, this interaction was not statistically significant (*β* = −9.8, *p* = 0.10). According to this model, fibrinogen levels increased significantly over time in both groups (*β* = 63.2, *p* < 0.001). Importantly, the mixed‐effects analysis identified that the intervention group experienced a significantly smaller increase in fibrinogen compared to the control group (interaction effect: *β* = −24.6, *p* = 0.011). Mixed‐effects analysis of ESR values indicated a significant increase over time (*β* = 11.2, *p* = 0.036), and the interaction between group and time was non‐significant (*β* = −6.9, *p* = 0.34).

### Effectiveness on post‐operative outcomes

VAS scores were similar between groups throughout the early post‐operative period (Days 0–3). Throughout the follow‐up period, there were no differences in knee circumference measurements, both before and after cryotherapy sessions, and for skin temperature measurements between groups in the affected knees across the cohorts (Table 3). The mixed‐effects model for VAS pain scores demonstrated significantly higher values before treatment compared to after treatment (*β* = 0.4, *p* = 0.004). This model revealed that the control group showed marginally higher baseline VAS scores (*β* = 0.4, *p* = 0.052). The mixed‐effects analysis found no significant interactions between group and day (*β* = −0.1, *p* = 0.10), group and before/after treatment (*β* = −0.2, *p* = 0.28), or in the three‐way interaction (*β* = 0.02, *p* = 0.88), suggesting similar pain response patterns in both groups.

No differences between groups at any time point were found for differences between the contralateral and the affected knee circumferences and skin temperature across the cohorts (Table 4). Skin temperature measurements were taken on both legs prior to each cryotherapy session to evaluate the residual effects of the previous day's treatment on the affected knee.

**Table 4:** Knee circumference and skin temperature differences between contralateral and affected across the cohorts.

	Cohort 1			Cohort 2			Differences	
	Median	SD	Q1–Q3	Median	SD	Q1–Q3	*p*	Effect size (*r*)
Swelling Day 0	−3	1.3	−4 to −2	−3	1.2	−3 to −1.8	0.25	0.11
Swelling Day 1	−4	1.4	−4.1 to −2.8	−3	1.4	−4 to −2	0.20	0.13
Swelling Day 2	−4	1.5	−4.5 to −3	−3.5	1.5	−4.5 to −2.5	0.93	0.00
Swelling Day 3	−3.5	1.6	−4.7 to −3	−4	1.7	−5 to −2.8	0.40	0.10
Swelling Day 20	−3	1.3	−4 to −2	−3	2.0	−4 to −2	0.91	0.01
Skin T° Day 0	0	0.4	−0.1 to 0.2	0	0.7	−0.4 to 0.1	0.13	0.15
Skin T° Day 1	−0.1	0.2	−0.3 to 0	−0.05	0.4	−0.4 to 0.1	0.57	0.05
Skin T° Day 2	−0.15	0.4	−0.5 to 0	−0.2	0.4	−0.4 to 0	0.71	0.03
Skin T° Day 3	−0.1	0.3	−0.3 to 0	0	0.3	−0.3 to 0.1	0.54	0.07

Abbreviations: 95% CI, 95% confidence interval; Q1, 1st quartile; Q3, 3rd quartile; SD, standard deviation.

By post‐operative Day 20, both groups achieved comparable functional outcomes for OKS and KSS, but a greater ROM in flexion was recorded for the intervention group (Table 3). The functional improvements with MCID across both cohorts are shown in Table 5.

**Table 5:** Functional improvement across both cohorts with the percentage of patients who reached the MCID thresholds.

	Cohort	*p*	Median difference	Median difference 95% CI	MCID	% Patients reaching MCID
KSS	Intervention	<0.001	19.5	[16.9–25.5]	4.5	94.7
KSS	Control	<0.001	20	[16.9–24.0]	4.1	93
OKS	Intervention	0.4	−1	[−2.5 to 1.5]	3.1	19.5
OKS	Control	0.50	−1	[−2.0 to 1.0]	2.4	30
ROM	Intervention	0.38	0	[−2.5 to 7.5]	5.8	28.6
ROM	Control	0.39	0	[−7.4 to 2.4]	5.9	20.9

Abbreviations: 95% CI, 95% confidence interval; KSS, Knee Society Score; MCID, minimally clinically important difference; OKS, Oxford Knee Score; ROM, range of motion.

A shorter time to reach the rehabilitation milestones to discharge was found in the intervention group (1.3 ± 0.4 vs. 1.2 ± 0.4 days, *p* = 0.007).

**Figure 4:**
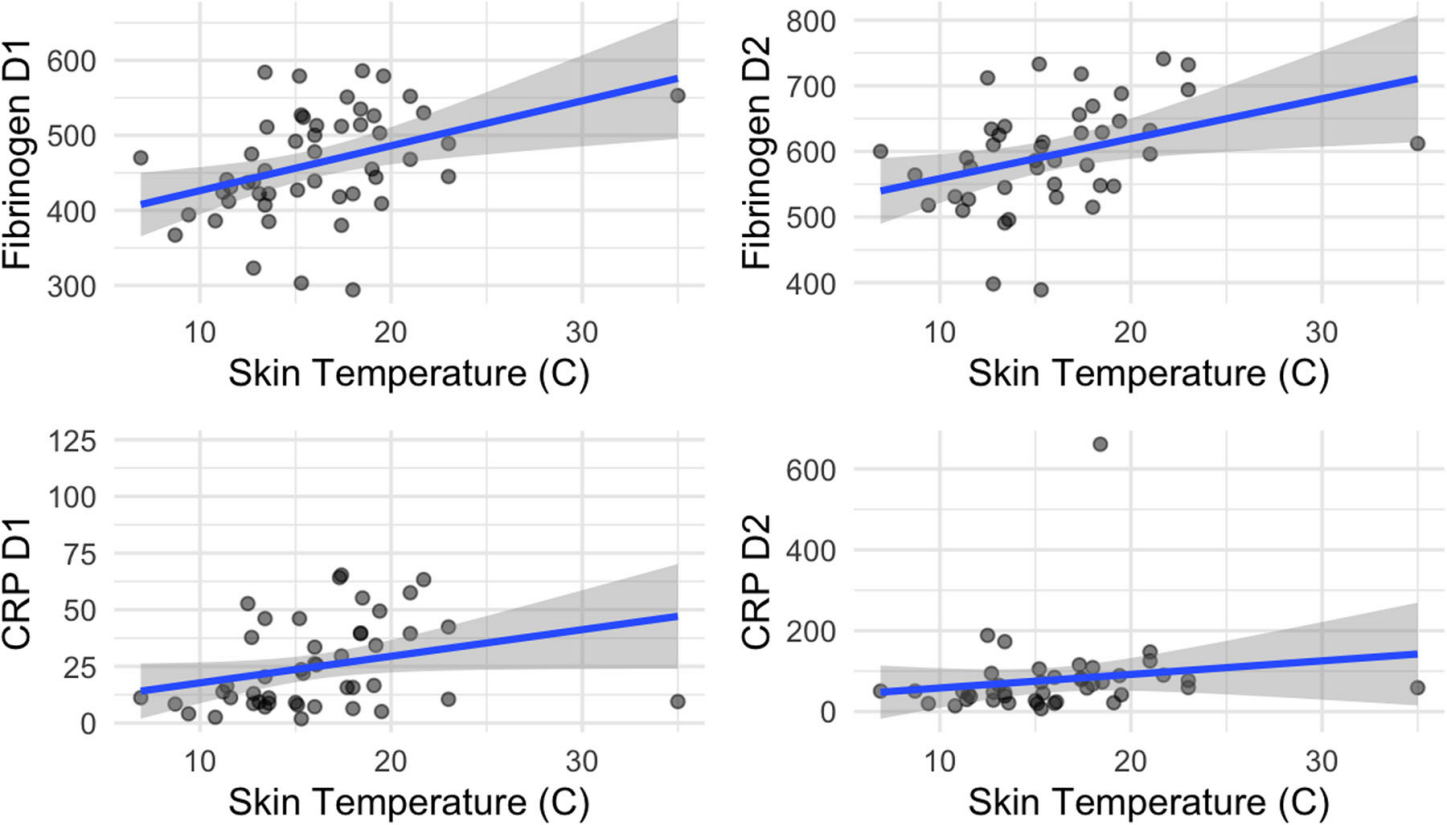
Correlation between skin temperature and clinical biomarkers. CRP, C‐reactive protein.

A strong positive correlation was found between skin temperature before skin incision during surgery and post‐operative inflammation parameters, specifically CRP (*r* = 0.3, *p* < 0.01) and fibrinogen (*r* = 0.4, *p* < 0.01) (Figure 4). No complications were reported.

## DISCUSSION

The most important finding of this study was that patients who received preoperative cryocompression therapy for 1.5 h before TKA showed lower early post‐operative inflammatory markers, a shorter timing to achieve rehabilitation milestones and greater flexion ROM at the first outpatient visit at 20 post‐operative days compared to those who received only post‐operative cryocompression therapy. Specifically, patients in the intervention group showed lower ESR values on both post‐operative Day 1 (*p* = 0.002) and Day 2 (*p* = 0.032), with a positive observed correlation between skin temperature before surgical incision and post‐operative inflammatory markers (CRP and fibrinogen). Additionally, the mixed‐effects analysis on ESR further strengthened the understanding of the differential inflammatory profile between groups. Fibrinogen showed baseline differences between groups, complicating direct comparisons. However, different increasing patterns across the groups when analyzing the change in fibrinogen levels from preoperative to post‐operative Day 2 were found, suggesting that preoperative cryotherapy did modulate the overall inflammatory response. The linear mixed‐effects analysis confirmed the significantly smaller increase in fibrinogen over time in the Intervention group demonstrating a clinically meaningful difference in the inflammatory response. The same analysis on CRP levels resulted in a significant time effect observed in CRP levels across both groups (*p* < 0.001), aligning with the expected inflammatory response following an acute stressor, such as the surgical intervention. Although a trend towards attenuated CRP elevation in the Intervention group compared to controls was observed, the statistical significance in this interaction was weak, suggesting that a wider sample size is needed to find a clinically meaningful effect on this acute‐phase protein. These different responses highlight the complex nature of the post‐surgical inflammatory cascade and suggest that preoperative cryotherapy may influence specific inflammatory pathways more than others. This variance in inflammatory marker response may also be attributed to the different kinetics of these biomarkers following surgical trauma.

Interestingly, despite the differences in inflammatory markers, no differences in the PROMs between the groups were found: both cohorts achieved comparable OKSS and KSS scores by Day 20. However, the intervention group showed greater flexion ROM (*p* = 0.001). This data is particularly important as ROM reflects both joint inflammation status and the functional status of the knee following TKA surgery [4, 20, 36, 37]. Moreover, patients undergoing primary TKA can expect clinically meaningful improvements in the first 6 months after surgery, regardless of BMI, age, American Society of Anesthesiologists class or timing of surgery, with relative minimum improvement in the early weeks following surgery [7, 10, 23, 29]. The improved ROM among intervention group patients suggests cryotherapy's potential benefits on joint recovery during the initial 20‐day post‐operative period.

The current findings revealed a modest but significant reduction in rehabilitation milestone achievement time in the intervention group (1.3 ± 0.4 vs. 1.2 ± 0.4 days, *p* = 0.007). While this 11.8% improvement in time efficiency may be small in absolute terms, its clinical significance should be considered within the context of an already optimized ERAS recovery protocol. Moreover, faster achievement of rehabilitation milestones may facilitate earlier hospital discharge, particularly relevant in the outpatient surgery [22, 25, 31].

Cryocompression therapy is a fundamental component of multimodal treatment following TKA, when implemented with appropriate equipment and protocols [32, 33, 34]. Both quantifiable outcomes and patient‐reported results clearly demonstrate the effectiveness of this approach [5, 17].

Previous research has documented that cryotherapy modifies synovial fluid composition by reducing inflammatory mediators and altering metabolite concentrations, resulting in both anti‐inflammatory and anti‐oxidative effects [6, 9, 30].

These results expand upon previous research demonstrating the benefits of post‐operative cryotherapy by suggesting that the timing of cryotherapy application may be important. The preoperative application appears to create a more favourable anti‐inflammatory environment for surgery, potentially supporting recovery within the first month of rehabilitation and suggesting a potential role for hypothermia in surgery, which needs further studies and wider sample sizes. This study represents the first investigation of preoperative cryocompression therapy in TKA patients. While post‐operative cryotherapy has been well‐studied, the preoperative application represents a novel approach within ERAS protocols that has not been previously evaluated in the literature. Despite evaluating a potentially subtle intervention (preoperative vs. only post‐operative cryotherapy), significant improvements were demonstrated in ⁠ROM at 20 days post‐operation, different markers of early post‐operative inflammation, and time to achieve rehabilitation milestones. In the context of an already optimized ERAS protocol, demonstrated by an all‐point median VAS scale of 0, achieving statistically and clinically significant differences represents a valuable finding, as it becomes increasingly challenging.

This study presents some limitations: the single‐centre design and the single‐cryotherapy device model used limited generalizability; blinding of both surgeons and patients was unfeasible due to the intervention nature, as patients would also be aware of the cooling treatment, and surgeons were aware of the treatment allocation. Moreover, there was a significant difference in gender distribution between groups (intervention: 60% women; control: 86% women; *p* = 0.003), which might have influenced our findings given that women may report different pain experiences, functional outcomes, and inflammatory responses after TKA, with potential variations in blood biomarker levels [8]. Additionally, the 20‐day follow‐up period limits our evaluation of longer‐term functional outcomes and complications, with findings primarily reflecting early post‐operative results. Finally, the lack of prior studies on preoperative cryotherapy in TKA required assuming a medium effect size for sample size calculation, which may have affected the study's statistical power to detect smaller but clinically relevant differences between groups. Although these results suggest preoperative cryocompression therapy could be a valuable addition to existing TKA protocols, the study's limitations indicate the need for larger multi‐centre trials to validate these findings and establish standardized protocols before widespread implementation.

## CONCLUSIONS

Preoperative cryocompression therapy before TKA reduces early post‐operative inflammation, accelerates rehabilitation milestones, and improves early ROM. These findings highlight a potential role for hypothermia in TKA surgery, warranting further investigations in larger studies.

## AUTHOR CONTRIBUTIONS

The authors would like to thank Giuseppe Cavallo for his valuable contributions to data preparation, data analysis, data interpretation and preparation of the first draft of the manuscript. Andrea Baldini contributed to the conception and design of the study. Material preparation and data collection were performed by Leonardo Pieri and Dimitri Bartoli. Data analyses were performed by Giuseppe Cavallo and Filippo Leggieri. Marco Ponti, Chiara Caparrini and Filippo Leggieri assessed the interpretation of the data results and supervised the study from inception. The first draft of the manuscript was written by Leonardo Pieri and Giuseppe Cavallo. Andrea Baldini and Filippo Leggieri reviewed the draft, and all the authors commented on previous versions of the manuscript. All the authors read and approved the final manuscript.

## CONFLICT OF INTEREST STATEMENT

The authors declare no conflicts of interest.

## ETHICS STATEMENT

The study adhered to the principles outlined in the Declaration of Helsinki. The trial protocol was approved by the Institutional Ethics Committee (Protocol No. 5/21/CTS). All patients provided informed consent before enrolment in the study.

## Supporting information


**Supporting information – CONSORT Checklist.** Reporting checklist for randomised trial. Based on the CONSORT guidelines.

## Data Availability

The data sets used and/or analyzed during the current study are available from the corresponding author on reasonable request.
